# Does Methylphenidate Cause a Cytogenetic Effect in Children with Attention Deficit Hyperactivity Disorder?

**DOI:** 10.1289/ehp.9866

**Published:** 2007-02-21

**Authors:** Susanne Walitza, Birgit Werner, Marcel Romanos, Andreas Warnke, Manfred Gerlach, Helga Stopper

**Affiliations:** 1 Department of Child and Adolescent Psychiatry and Psychotherapy and; 2 Department of Toxicology, University of Würzburg, Würzburg, Germany

**Keywords:** ADHD, cytogenetic effects, methylphenidate, micronuclei, psychostimulants

## Abstract

**Background and objective:**

Attention deficit hyperactivity disorder (ADHD) is the most common psychiatric disorder in children and adolescents (6–12% affected). Treatment with methylphenidate (MPH) in the United States has increased to a current prescription rate of > 5 million per year. However, a 2005 study by El-Zein and co-workers [Cancer Lett 230:284–291] reporting a 3-fold increase in genomic damage in all 12 analyzed children after 3 months of therapy with MPH resulted in much concern about potential carcinogenic effects. Here we provide new information concerning the cytogenetic effect of MPH in children.

**Design, participants, and methods:**

In a prospective study, we analyzed the genomic damage in children with ADHD (initial sample size 38 children) before and 1 (30 children), 3 (21 children), and 6 (8 children) months after initiation of MPH therapy. In addition, we investigated a group of 9 children receiving chronic MPH therapy. Patients were recruited within a study of our Clinical Research Group on ADHD in the Department of Child and Adolescent Psychiatry and Psychotherapy of the University of Würzburg. Assessment and treatment of patients were performed during inpatient or outpatient health care. The measure for genomic damage was the frequency of micronuclei, a subset of chromosomal aberrations, in peripheral lymphocytes.

**Results:**

MPH treatment resulted in no significant alteration in the micronucleus frequency.

**Conclusions:**

Because the findings published in 2005 by El-Zein and co-workers could not be replicated, the concern regarding a potential increase in the risk of developing cancer later in life after long-term MPH treatment is not supported.

Attention deficit hyperactivity disorder (ADHD) is the most common psychiatric disorder in children and adolescents, with 6–12% being affected worldwide according to the *Diagnostic and Statistical Manual of Mental Disorders*, 4th text revision (DSM-IV-TR 2000; see also Biederman and Faraone 2005). Methylphenidate (MPH), a central nervous system stimulant, is the most frequently prescribed drug for the symptomatic treatment of ADHD and has consistently shown efficacy and safety. Although MPH was introduced about 50 years ago, no conclusive data are currently available to assess the long-term benefits and risks. A recent report (El-Zein et al. 2005) on cytogenetic effects observed in peripheral lymphocytes (PBL) from 12 ADHD children treated for 3 months with MPH raised questions about the genetic toxicity of this drug. On average, 3.0-, 4.3-, and 2.4-fold increases were found in chromosome aberrations (12 cases), sister chromatid exchanges (11 cases), and micronuclei frequencies (11 cases), respectively. The authors (El-Zein et al. 2005) cautioned that “the data should be replicated and expanded” but also stated that “the lack of research on long-term effects of methylphenidate use in humans warrants great concern.” This great concern arose because cohort studies have shown an association between the frequency of cells with structural chromosomal aberrations or micronuclei in peripheral blood lymphocytes and cancer risk (Bonassi et al. 2004, 2006; Hagmar et al. 2004). Considering the extremely high current estimated prescription rate of MPH of over 5 million per year in the United States (Physician Drug and Diagnosis Audit 2003), a thorough investigation of the safety of this important medication for children with ADHD is essential.

Using a standard genotoxicity test battery, Teo et al. (2003) evaluated the activity of MPH in the bacterial reverse mutation assay and mouse lymphoma mammalian mutation assay with and without metabolic activation, and in a bone marrow micronucleus test in male and female CD-1 mice. Although substance-associated toxicity was observed in the mammalian tests, no mutagenic or clastogenic effects were induced. This was recently further supported by negative genotoxicity findings in the *in vitro* human lymphocyte chromosomal aberration assay and the mouse bone marrow micronucleus test (Suter et al. 2006).

Epidemiologic data are limited to one report of a cohort study of 143,574 patients; the researchers screened MPH, among other prescription drugs, for possible carcinogenicity and found no increase in cancer (Selby et al. 1989). However, the small sample size of 529 MPH-treated patients (15 cancer cases instead of 32.7 expected cases) and the short observation period of up to 15 years limit the interpretation. According to recent overviews responding to the findings of elevated genomic damage in children (Holtmann et al. 2006; Preston et al. 2005), MPH was not found to be carcinogenic in a long-term rat bioassay, but yielded an increase in hepatic neoplasms in B6C3F_1_ mice at the highest test dose of 56–66 mg/kg body weight/day, which is about 60-fold above the recommended doses for ADHD patients (Taylor et al. 2004). Because of these hepatic neoplasms, MPH was judged by the National Toxicology Program (NTP) of the National Institute of Environmental Health Sciences to yield “some evidence of carcinogenic activity” (NTP 1995).

The aim of this study is to provide new data on the potential mutagenicity of MPH in children. We assessed the cytogenetic effects of MPH treatment by evaluating the frequency of micronuclei, a subgroup of chromosomal aberrations, in ADHD children. We present data on the results before and 1 month (30 children), 3 months (21 children), and 6 months (8 children) after initiation of MPH treatment. Additionally, we analyzed 9 patients who had been medicated with MPH between 6 and 24 months.

## Methods

### Participants

The study was approved by the ethics committee of the University of Würzburg (study no. 140/03), and written informed parental consent was obtained for all patients before participation in the study.

Children with ADHD and no previous medication were recruited between May 2005 and April 2006. The patients were characterized by a team of consultant child and adolescent psychiatrists at the Department of Child and Adolescent Psychiatry and Psychotherapy of the University of Würzburg. The patients were assessed and treated during inpatient or outpatient health care; semistructured interview and the Schedule for Affective Disorders and Schizophrenia for School-Aged Children (K-SADS) (Kaufman et al. 1997) were used as diagnostic inventories. The recruitment was done within a study of our Clinical Research Group on ADHD with the objective of family-based association and genome-wide linkage studies.

Exclusion criteria were current smoking, current infection or an infection in the 14 days before blood sampling, or extreme food patterns (e.g., vegans) of the children. Psychiatric diagnoses such as anorexia nervosa, schizophrenia, any pervasive developmental disorders, neurologic disorders such as epilepsy, a history of any acquired brain damage, or evidence of a fetal alcohol syndrome, premature deliveries and/or maternal reports of severe prenatal, perinatal, or postnatal complications, as well as severe diseases were also regarded as exclusion criteria.

We investigated an additional sample consisting of nine children, who had been medicated with MPH for more than 6 months (chronic treatment). Four of them had received comedications (three risperidone and one valproic acid) before the beginning of the study. Except for the medication, the same recruitment and exclusion criteria were applied. The MPH treatment (organized by sex) for the prospective study and the chronic treatment group is given in the tables.

### Group description and missing data of the prospective investigation

All children fulfilled the diagnostic criteria for ADHD according to DSM-IV-TR (2000) and were completely drug naive before being enrolled in the study. Of the 38 patients who were identified as potential participants, 8 patients (group A) were not medicated with MPH or did not want to participate further in the study. Two patients withdrew because of nonresponse to MPH and change of treatment to amphetamine and atomoxetine, respectively, and 7 patients were lost at follow-up. These circumstances both occurred after the 1-month visit. Therefore, 9 patients (group B) could be placed in the 1-month follow-up, 13 patients (group C) could be included in the 1- and 3-month follow-up, and 8 more patients were analyzed after 1, 3, and 6 months (group D). Thus the groups A, B, C, and D represent patients from whom we could collect one, two, three, and four blood samples, respectively, during the course of the study (Tables 1 and 2).

### Collection of blood samples

Blood samples (7.5 mL each) were collected 1 day before, and 1, 3, and 6 months after MPH treatment for micronucleus analysis. From each participant of the chronic treatment group, one blood sample was analyzed. Blood samples were taken via an in-dwelling cannula and collected in coded tubes containing heparin. When blood samples were collected before 1700 hr, the samples were transported at room temperature to the nearby Department of Toxicology, University of Würzburg, for immediate isolation of peripheral blood mononuclear cells (PBMCs); when samples were taken after 1700 hr, they were stored at room temperature pending isolation of PBMC the following morning.

### Cell isolation

PBMCs were isolated by standard density gradient centrifugation using FicoLite H (Linaris-H, Wertheim, Germany). In brief, the blood was layered (1:1) onto FicoLite H (Linaris) and centrifuged at 1,600 rpm (370 × *g*) for 30 min at room temperature. The PBMC layer was removed and the cells were washed twice (1,300 rpm; 250 × *g*; 10 min) with RPMI-1640 (Sigma-Aldrich Chemie GmbH, Steinheim, Germany).

### Culture conditions

PBMCs were cultured at a cell density of 1 Mio (millions)/mL in 5 mL of culture medium at 37°C in a humified, 5% carbon dioxide incubator. The culture medium consisted of RPMI-1640, supplemented with 15% fetal bovine serum, 2 mM l-glutamine, 1 mM Na-pyruvate, nonessential amino acids, and antibiotics (penicillin/streptomycin/tylosin). To stimulate the lymphocytes for proliferation, PHA (phytohemagglutinine; final concentration 10 μg/mL) was added.

### Micronucleus assay

The micronucleus scoring was carried out by a single scorer six times for each sample in a blinded manner. Because DNA damage can be expressed as micronuclei only after going through mitosis, the analysis was limited to actively dividing lymphocytes. These were identified as binucleated cells on addition of the cytokinesis-inhibitor cytochalasin B (final concentration 5 μg/mL). Cytochalasin B was added to the cultures 44 hr after stimulation with PHA. At 72 hr after culture initiation, the cells were spun on glass slides with a cytocentrifuge (Cytospin 3; Shandon/Thermo, Dreieich, Germany; 1,000 rpm; 145 × *g*, 5 min). Subsequently, fixation in cold (−20°C) methanol was performed for at least 2 hr. Slides were stored at −20°C in sealed boxes. Before evaluation, slides were stained with acridine orange (0.00625% in Sorensen buffer, w/v, pH 6.8) for 5 min, and washed twice in Sorensen buffer [67 mM Na_2_HPO_4_/KH_2_PO_4_ (pH 6.8)]. Sample preparation (cell culture, slide preparation) was limited to two laboratory members, staining of slides to one. From each blood sample, 6,000 binucleated lympocytes (6 slides, 1,000 cells per slide) were screened for the presence of micronuclei with fluorescence microscopy at 400× magnification. The scoring criteria corresponded to those described by Fenech et al. (2003).

In addition, from 6,000 cells of each sample, we determined the cytokinesis block proliferation index (CBPI) [(number of mononucleate cells + 2× number of binucleate cells + 3× number of multinucleate cells)/ (sum of mononucleate, binucleate, and multinucleate cells)].

### Statistics

Data are shown as mean ± SD of micronucleated cells per 1,000 binucleated cells. Microsoft Excel was used to organize data. WinStat (demo version; R. Fitch Software, Bad Krozingen, Germany; Statistics for Excel, Microsoft Corporation, Redmond, WA, USA) was applied for calculation of correlations (Pearson). Because data were not strictly normally distributed (as analyzed by WinStat) we applied both the paired *t*-test and Wilcoxon test for comparison of each sampling time with values before initiation of therapy. An overall analysis and an analysis of the most informative subgroup across three sampling times (before, 1 month, 3 months) were performed with Kruskal-Wallis. Independent groups were compared with the two-sample *t*-test and the two-sample Wilcoxon test. A *p*-value of ≤ 0.05 was considered significant.

## Results

**Figure 1:**
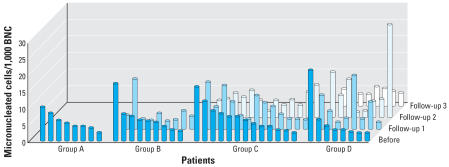
Individual frequencies of micronucleated peripheral lymphocytes of children with ADHD before and after MPH treatment at three follow-up intervals. BNC, binucleated cells. In group A, all children were included for whom only a basal value (before start of medication) was obtained. The groups B, C, and D included patients for whom values after 1 month (follow-up 1), 3 months (follow-up 2), and 6 months (follow-up 3) were obtained. Data were sorted according to the heights of the column for the basal values.

The mean (± SD) age of the children in the initial study group (*n* = 38; groups A–D, Tables 1, 2; Figure 1) was 10.0 ± 2.8 years (range, 4.9–17.0 years). The sample included 29 male and 9 female children (all of German origin). The mean age of the 21 patients from whom at least three blood samples were collected (groups C + D; Tables 1, 2; Figure 1), was 9.7 ± 2.8 years (range, 4.9–17.0 years). The average of children’s body weight was 34.5 ± 13.5 kg (range, 23.6–82.0 kg). During the first month, the children were medicated with an individual dose of MPH, ranging from 5 to 40 mg/day, equivalent to a mean of 0.54 mg/body weight/day, and during the subsequent 3 months with a dose of MPH ranging from 15 to 45 mg/body weight/day, equivalent to 0.74 mg/body weight/day. Sex, individual MPH doses, and pharmaceutical preparation (short-acting, long-acting) are given in Table 1. Comparable to our investigation, in the study of El-Zein et al. (2005) the patients received therapeutic dosages ranging from 20 to 54 mg/day after 3 months.

In our chronic treatment group (Table 3), consisting of nine boys with long-term medication of MPH (6 months to 2 years) the mean age was 11.2 ± 2.8 years (range, 7.1–16.0 years). All of them had been medicated with a dose ranging from 15 to 60 mg/day. The average body weight was 45.5 ± 16.2 kg (range, 22.8–77.1 kg), and the average dose of MPH was equivalent to a mean of 0.83 mg/body weight/day. Three of these patients had additionally received risperidone (range, 0.5–1.5 mg/day) and one of them valproic acid (1,500 mg/day) for longer than 6 months.

From the prospective study, 38 pretreatment blood samples were available for micronucleus analysis in binucleated lymphocytes (groups A–D; Table 2, Figure 1). In untreated children we determined 6.1 ± 4.3/1,000 binucleated cells. There was no correlation between basal micronucleus numbers and sample admission date (*r* = −0.22; *p* = 0.10), suggesting no seasonal influences on micronucleus numbers. Within this age group, donor age did not correlate with micronucleus frequency (*r* = 0.02; *p* = 0.45). Because of limited sample size (nine female patients), we performed no analysis for influence of sex.

Figure 1 and Table 2 give the number of micronucleated cells before and after MPH treatment at three different follow-up time points. The micronucleated cell frequencies at each follow-up compared with the baseline value did not demonstrate any significant increase (paired *t*-test and Wilcoxon test). Analysis of all groups with Kruskal-Wallis yielded no significant alterations. Table 2 also indicates that there was no significant alteration (*t*-test and Wilcoxon test) in the cell proliferation capability in response to phytohemagglutinine stimulation *in vitro* (CBPI) after initiation of MPH therapy.

In addition, we gathered samples (one time point) from children who had received MPH for at least 6 months (6 months–2 years of chronic treatment). This group of nine children (Table 2) did not show a significantly different (*p* = 0.49/0.69; *t*-test/Wilcoxon; two sample tests performed separately) mean micronucleated lymphocyte frequency compared with the pre-treatment mean of our study group.

In addition to the assessment of micronucleus numbers, we measured safety by analyzing changes in vital signs, electrocardiograms, and clinical laboratory values such as white and red blood count, electrolytes, and transaminases according to the recommendations for MPH therapy (Warnke and Walitza 2004). No abnormal parameters were observed except slightly reduced values of total iron, without signs of hypochromic microcytic anemia, which occurred in some patients before and during the treatment, independent of micronucleus deviation. We could not detect reasons for increased micronucleus frequencies of individual children at isolated time points such as pathologic laboratory parameters, eating behavior, weight, or dose per weight. We reconsulted all patients with micronucleus frequencies of ≥ 10/1,000 binucleated cells to ascertain whether they had been exposed to radiation, such as medical X-ray exposure or cosmic radiation within the preceding 2 years. Because only one patient reported one flight 2 years before the study, one patient had been hiking higher than 3,000 m, and another patient had one maxilla X ray for getting a brace, radiation exposure does not explain these isolated elevated micronucleus frequencies at single time points.

## Discussion

In this study we did not find any alteration in the number of micronucleated cells after MPH treatment at three follow-up intervals (up to 6 months). These results suggest no induction of genomic damage and are in contrast to a recent report by El-Zein et al. (2005) showing elevated genomic damage 3 months after initiation of MPH therapy using three cellular genotoxicity end points—frequency of micronucleus formation, chromosomal aberrations, and sister-chromatid exchanges (SCE).

A micronucleus is formed during cell division, expressing previously induced chromosomal damage, and contains chromosomal fragments and—less frequently—whole chromatids or chromosomes. SCE analysis detects exchanges between sister chromatids harboring identical genetic information, thus not directly representing a mutagenic effect. Although chromosomal aberration studies are very common in human genetic analysis and cancer research, micronuclei are the cytogenetic end point most often used for current human exposure biomonitoring studies. For example, HUMN (Human MicroNucleus; http://ehs.sph.berkeley.edu/holland/humn/) is an international collaborative project of > 40 laboratories from all over the world documenting micronucleus frequencies in human populations. HUMN is currently focusing on human lymphocytes and exfoliated epithelial cells as biomonitors for exposure to toxic substances, and as potential predictors of adverse health effects (Fenech et al. 1999). Recently, Bonassi et al. (2006) found that an increased micronucleus frequency in peripheral blood lymphocytes predicts the risk of cancer in humans.

El-Zein et al. (2005) reported an average micronucleus frequency of 3.55 ± 0.68/1,000 binucleated cells in drug-naive patients, whereas we found 6.1 ± 4.3 micronucleated cells/1,000 in this study. As with El-Zein et al., some researchers prefer to score the number of micronuclei per 1,000 cells rather than the number of micronucleated cells, as recommended for genotoxicity testing in the Organisation for Economic Co-operation and Development (OECD) guideline draft (OECD 2006). Because cells with more than one micronucleus are rare, the difference between these variants is small, and the number of micronuclei would be higher than the number of micronucleated cells. In a meta-analysis aimed at providing reference values for basal micronucleus frequencies (no exposure to genotoxic agents, no disease), data from 13 publications with micronucleus data of children (440 children 0–19 years of age) gives an overall mean of 4.48 ± 0.66 micronuclei/1,000 binucleated cells (Neri et al. 2005). Based on the data collection from 12 laboratories for a pooled analysis of individual data (332 referents, 0–18 years of age; data from HUMN database) an overall mean ± SE of 5.23 ± 5.07 micronuclei/1,000 binucleated cells was calculated (Neri et al. 2005). Specifically, the age groups of 5–9 and 10–14 years had pooled estimated means of 5.62 and 6.02, respectively. Thus, our data (mean age, 10.0 years) are in excellent agreement with the published literature.

Searching for possible explanations for the difference between our study and that of El-Zein et al. (2005), we are not aware of variations between the methods and procedures; El-Zein et al. (2005) did not describe methods in detail because of space limitations, so they denoted them as “standard procedures” in a later comment (El-Zein et al. 2006). In that comment, they excluded changes in the patients’ health, environment, diet, or lifestyle as reasons for the observed elevation in the level of cytogenetic damage. Seasonal influences could be excluded in our database; it is not known whether this was also possible in their study. Differences concerning doses of MPH or type of pharmaceutical preparation, potentially increasing the contact time between MPH and peripheral lymphocytes, are not apparent between both studies, but El-Zein et al. (2005) do not describe individual dosage and pharmaceutical preparation of the medication.

Although there is no evidence of cytochrome P450 (CYP450)-dependent reactions in the metabolism of MPH in humans, Le Nedelec and Rosengren (2002) have found that MPH decreased total hepatic CYP450 in the mouse and altered the catalytic activity and/or the polypeptide levels of CYP1A, CYP2E1, and CYP3A. Thus, CYP450 polymorphisms may influence the effects of MPH. Because polymorphisms are not distributed equally among populations worldwide, we cannot exclude different extends of polymorphisms between the El-Zein study (2005) and ours. This presumption is supported by the fact that the investigated group of El-Zein et al. consisted of patients with different ethnicities (six Caucasians, four African Americans, and two Hispanic children), whereas our sample includes only patients of German origin.

Based on our results, concern about a potential increase in the cancer risk later in life after long-term MPH treatment needs to be reconsidered. This is also supported by the fact that our “chronic treatment” group did not show a significant difference in micronucleated cells compared with the pretreatment values of the study group. Nonetheless, the widespread use of MPH still suggests a need for further research on this important topic, including extension of this study to examine potential long-term effects.

## Figures and Tables

**Table 1:** Individual dosage of MPH analyzed after 1, 3, and 6 months for all children of groups B, C, and D.

	Dosage of MPH (mg/day)		
Sex	1 month	3 months	6 months
Group B			
F	15		
M	40		
M	30		
M	20		
M	5		
M	10		
M	20 long-acting		
F	18 long-acting, 5 short-acting		
M	10		
Group C			
M	20	30	
F	10	20	
M	30 long-acting	40 long-acting	
M	40 long-acting	40 long-acting	
M	18 long-acting	36 long-acting	
M	20	30	
M	15	20	
M	15	15	
F	20	30	
F	10	20	
M	15	40	
M	5	20	
M	18	18	
Group D			
F	15	20	25 long-acting
M	20	20	30
M	20	20	30
M	10	15	15
F	36 long-acting	36 long-acting	54 long-acting
M	20	20	20
F	15	20	20
M	20	25	20 long-acting

Abbreviations: F, female; M, male. Unless otherwise noted, the short-acting pharmaceutical preparation has been given.

**Table 2:** Individual frequencies of micronucleated lymphocytes of children with ADHD before and after MPH treatment at three follow-up intervals.

	Before treatment		After 1 month		After 3 months		After 6 months	
Group (no. of subjects)	Mn-Cells	CBPI	Mn-Cells	CBPI	Mn-Cells	CBPI	Mn-Cells	CBPI
A (8)	10.00 ± 2.00	1.73						
	8.00 ± 4.73	1.73						
	5.83 ± 0.75	1.70						
	5.00 ± 0.89	1.69						
	4.00 ± 2.97	1.75						
	4.00 ± 2.00	1.73						
	3.67 ± 0.82	1.69						
	2.00 ± 0.89	1.88						
B (9)	17.00 ± 4.69	1.82	4.00 ± 1.79	1.79				
	7.83 ± 4.12	1.70	15.00 ± 6.13	1.74				
	7.00 ± 1.55	1.74	3.00 ± 0.89	1.85				
	6.00 ± 3.58	1.92	2.50 ± 0.84	1.56				
	5.67 ± 1.21	1.63	4.67 ± 1.03	1.63				
	5.33 ± 2.07	1.78	2.17 ± 1.33	1.69				
	4.00 ± 1.29	1.77	2.67 ± 0.82	1.78				
	3.00 ± 0.89	1.58	5.33 ± 1.03	1.67				
	2.67 ± 0.82	1.77	3.67 ± 0.82	1.68				
C (13)	16.00 ± 4.34	1.88	14.00 ± 2.37	1.81	5.00 ± 1.78	1.75		
	11.67 ± 4.08	1.70	6.67 ± 1.63	1.68	5.50 ± 1.38	1.66		
	8.83 ± 2.32	1.76	13.00 ± 2.53	1.74	7.33 ± 2.50	1.76		
	8.00 ± 0.89	1.69	8.17 ± 3.60	1.74	6.00 ± 1.67	1.70		
	7.00 ± 2.19	1.67	4.00 ± 3.03	1.75	8.00 ± 1.79	1.84		
	7.00 ± 1.41	1.79	3.00 ± 2.53	1.72	5.17 ± 2.32	1.82		
	6.00 ± 1.27	1.77	10.00 ± 2.83	1.85	1.67 ± 1.03	1.72		
	4.83 ± 2.14	1.75	7.83 ± 2.32	1.80	5.00 ± 2.10	1.78		
	4.00 ± 1.27	1.76	6.67 ± 1.03	1.85	3.67 ± 1.21	1.92		
	4.00 ± 2.00	1.73	4.33 ± 1.21	1.66	5.33 ± 1.37	1.66		
	3.00 ± 3.03	1.74	3.00 ± 1.55	1.73	3.67 ± 1.63	1.69		
	2.83 ± 2.14	1.75	3.00 ± 1.27	1.84	7.67 ± 2.94	1.73		
	2.00 ± 0.89	1.80	2.83 ± 1.47	1.85	9.67 ± 4.41	1.81		
D (8)	21.00 ± 5.22	1.76	10.00 ± 5.37	1.74	13.67 ± 3.50	1.85	5.00 ± 0.89	1.71
	6.17 ± 1.17	1.81	5.33 ± 1.03	1.81	6.50 ± 1.38	1.84	5.67 ± 0.82	1.78
	4.17 ± 1.47	1.73	2.00 ± 2.28	1.77	11.50 ± 4.85	1.77	3.50 ± 1.64	1.80
	3.17 ± 2.86	1.65	3.00 ± 1.41	1.74	1.50 ± 1.05	1.67	3.33 ± 0.52	1.67
	3.00 ± 1.79	1.78	16.00 ± 2.19	1.85	2.00 ± 1.41	1.72	5.67 ± 1.37	1.79
	2.67 ± 1.75	1.74	3.00 ± 1.27	1.72	4.83 ± 2.48	1.78	5.17 ± 1.17	1.74
	2.00 ± 0.63	1.80	8.17 ± 3.76	1.78	5.50 ± 2.34	1.68	4.33 ± 1.03	1.64
	2.00 ± 0.63	1.69	2.00 ± 1.27	1.70	27.83 ± 3.71	1.78	3.67 ± 0.82	1.70
Total no. of patients	38	38	30	30	21	21	8	8
Mean ± SD	6.06 ± 4.29	1.75 ± 0.07	5.97 ± 4.12	1.76 ± 0.08	7.00 ± 5.65	1.76 ± 0.07	4.54 ± 0.96	1.73 ± 0.06
*p*-Value (*t*-test/Wilcoxon)			0.75/0.75	0.53/0.41	0.60/0.96	0.61/0.78	0.67/0.55	0.54/0.81

Values for micronucleated lymphocytes (Mn-Cells) are mean ± SD from 6 analyses of 1,000 binucleated lymphocytes. The cytokinesis block proliferation index (CBPI) from 6,000 cells is indicated for each time point. Significance was analyzed using the paired *t*-test and the Wilcoxon test to compare data from each time point with the same individuals before therapy initiation.

**Table 3:** Individual frequencies of micronucleated lymphocytes of children with ADHD who had received chronic MPH treatment for at least 6 months.

Treatment duration (months)	Sex	Dosage of MPH (mg/day)	Mn-Cells	CBPI
13	M^a^	60	3.67 ± 0.82	1.70
6	M^b^	35	4.00 ± 0.89	1.67
8	M	45	5.83 ± 1.47	1.73
24	M^c^	25	2.00 ± 1.79	1.63
15	M	54 long-acting	5.33 ± 1.21	1.60
11	M	25	7.67 ± 0.82	1.67
6	M	40	1.83 ± 1.33	1.62
12	M^d^	40	8.83 ± 1.47	1.79
24	M	15	6.00 ± 0.89	1.75
Total no. of patients			9	9
Mean ± SD			5.02 ± 2.38	1.69 ± 0.06

M, male. Values for micronucleated lymphocytes (Mn-Cells) are mean ± SD from 6 analyses of 1,000 binucleated lymphocytes. The cytokinesis block proliferation index (CBPI) from 6,000 cells is indicated.

a Risperidone: 0.5 mg/day.

b Risperidone: 1 mg/day.

c Risperidone: 1.5 mg/day.

d Valproic acid: 1,500 mg/day.
